# Relationship between Physical Fitness and Academic Performance in University Students

**DOI:** 10.3390/ijerph192214750

**Published:** 2022-11-10

**Authors:** Laura Redondo-Flórez, Domingo Jesús Ramos-Campo, Vicente Javier Clemente-Suárez

**Affiliations:** 1 Faculty of Sport Sciences, Universidad Europea de Madrid, 28670 Madrid, Spain; 2 Department of Health and Human Performance, Universidad Politécnica de Madrid, Martin Fierro Street, s/n, 28040 Madrid, Spain

**Keywords:** blood pressure, body composition, grade, maximum oxygen uptake, sleep patterns

## Abstract

Several studies involving students have been developed with the objective to analyse the influence of different factors on academic performance. Nevertheless, all these studies were focused on stress and sympathetic modulation response instead of contemplating other physiological parameters that may affect academic performance. The aim of the present study was to analyse body composition, cardiovascular, sleep habits and physical activity factors related to the academic performance of university students. Two hundred and sixty-one students with bachelor’s degrees in physical activity and sports science participated in the present study (age: 22.49 ± 3.84 years; weight: 73.94 ± 11.4 kg; height: 176.28 ± 7.68 cm; 87.7% males). Participants were divided into two groups according to their academic performance: low academic performance group (LAPG) and high academic performance group (HAPG). Body composition, blood pressure, physical activity and sleep habits were measured, and maximum oxygen uptake was estimated by the Cooper’s 12 min run test. The results show that students with a high academic performance presented a higher VO_2_ max than the LAPG (LAPG = 40.32 ± 6.07; HAPG = 47.91 ± 6.89 mL/kg/min; *p* < 0.001), as well as lower diastolic blood pressure (LAPG = 72.44 ± 14.27; HAPG = 67.48 ± 13.50 mmHg; *p* < 0.01) and insomnia levels caused by breathing problems (LAPG = 0.37 ± 0.8; HAPG = 0.13 ± 0.42 a.u.; *p* = 0.046). Therefore, we found a relevant association between academic performance and VO_2_ max, diastolic blood pressure, and insomnia caused by breathing problems. These results highlight the importance of applying different programmes that may improve these factors, especially those related to physical activity and sleep habits in order to improve academic achievement.

## 1. Introduction

Academic performance could be a useful tool to evaluate learning in a university environment. In this context, several factors have been highlighted as important determinants of academic performance. Firstly, physical activity is thought to be a key element that may have a great impact on academic performance, since previous researchers indicated an improvement in different cognitive skills, such as execution, decision, perception, concentration and memory, which would aid regular activity practice [1,2,3]. Furthermore, previous researchers found a positive correlation between vigorous physical activity and higher academic performance [4]. Secondly, stress has also been highlighted as an important component that could have a negative impact on academic performance. Previous research described how acute stress led to a reduced blood flow in the prefrontal cortex, triggering a reduction in oxygen and nutrients in this area, causing concentration difficulties, lower memory potential and more decision making [5,6].

Additionally, stress compromises synaptic efficacy as well as cortical plasticity [7], which might hinder the learning process. Regarding stress, anxiety is also associated with academic achievement, since previous researchers found a negative association between high scores in trait anxiety (a measure of the anxiety levels) and academic performance in young students [8], showing that students with higher levels of anxiety have a worse academic performance. In this context, with the objective to determine anxiety levels and their influence on psychophysiological response, several authors measured heart rate in order to evaluate the sympathetic modulation of students in different academic fields [9,10,11]. High anxiety levels are related to autonomic activation [10], suggesting that greater anxiety levels led to higher autonomic modulation and lower academic achievement. Thirdly, cultural context may also have a great impact on learning since it has been found that students who work and study simultaneously or who live with their parents were more likely to abandon their academic studies [12,13]. 

On the contrary, some researchers found that family members provided both affective and economical support for students; students who had a higher perception of family support were more focused on achieving their academic objectives [14,15]. Regarding migration issues, previous studies described how migrant students who had fewer difficulties facing a host culture had more opportunities to earn better academic achievements [16]. Finally, another key factor that might affect academic performance is body mass index (BMI). Previous researchers found a negative association between high values of BMI and worse academic performance [17,18]. These high values of BMI could also be related to decreased physical activity, longer screen time or playing videogames [19,20,21], as well as unhealthy nutritional habits, including the consumption of sugary beverages and high-calorie foods [22]. According to these findings, nutrition is also a considerable factor that may compromise BMI and, consequently, academic performance.

In order to consider the importance of physical activity and its impact on academic performance, previous researchers indicated the presence of a substance that might improve academic achievement. Recent literature suggested the presence of a brain-derived neurotrophic factor (BDNF), whose synthesis is improved by cardiorespiratory fitness and whose activity could affect academic performance [23,24]. BDNF plays an important task in different brain functions and processes [25], such as providing synaptic plasticity in the hippocampus, modulating the creation of neuronal circuits, influencing emotional decision-making performance and helping to establish long-term memory [26,27,28]. Furthermore, lower levels of BDNF have been found in obese children and adolescents [29,30], suggesting the possibility of reduced academic performance. Additionally, previous researchers found a positive association between high academic performance and healthy physical condition, measured through different physical tests, such as short abs, long jump to feet together, forward trunk flex, elbows flexo-extension and the course Navette test. Students who had greater academic performance showed higher scores in these tests [31].

Several studies on students have been developed with the objective to analyse the influence of different factors on academic performance. Previous researchers evaluated in school students the impact of age, weight, body mass index and anxiety in academic grades, showing a negative correlation between these factors and academic performance [32]. In children of North African descent, the influence of nationality on autonomical nervous system stress markers, physical activity levels and academic performance was analysed, although without showing the differences between these children and their non-immigrant counterparts [33]. For university students, the psychophysiological stress response and general stress associated with writing a final dissertation in either their native language or a non-native language might affect cortical arousal and academic achievement, regardless of the psychophysiological response to different languages, which is increased in any case [34,35]. Nevertheless, all of these studies strongly focused on stress and sympathetic modulation response instead of contemplating other physiological parameters that may affect academic performance. The aim of the present study was to analyse body composition, cardiovascular function, sleep habits and physical activity factors related to the academic performance of university students. The initial hypothesis was that high-academic-performance students would present lower BMI and blood pressure levels, as well as having a greater physical activity and better sleep habits than students with lower academic performance.

## 2. Materials and Methods

### 2.1. Design

A descriptive and non-experimental study was performed. The study analysed anthropometric and physiological variables, physical activity and sleep quality habits and academic performance in exercise physiology matter in university students. 

### 2.2. Participants

Two hundred and sixty-one students with bachelor’s degrees in physical activity and sports science voluntarily participated in the present study (age: 22.49 ± 3.84 years; weight: 73.94 ± 11.4 kg; height: 176.28 ± 7.68 cm; 87.7% males and 12.3% females). All of them were students of the subject “exercise physiology” in this study. To achieve the main objective of the present research, participants were divided in two groups of equal size according to their academic performance (percentile 50). The low academic performance group (LAPG: *n* = 130) included scores between 0.2 and 5.0 of 10, and the high academic performance group (HAPG: *n* = 131) included scores between 5.0 and 9.4 of 10. Prior to the test, the procedures of the study were explained to all subjects, and they provided signed informed consent following the Helsinki Declaration (as revised in Brazil, 2013). All the data were anonymously collected, and the procedure was approved by the European University of Madrid Ethical Committee (CIPI/18/074).

### 2.3. Procedures

All measurements were taken one week before the experiment and the end of the academic year. All the tests were completed in one testing session. During the testing session, anthropometric variables, blood pressure, physical activity questionnaire, Pittsburg sleep quality questionnaire [36], and Cooper’s 12 min run test [37] were performed. 

When participants arrived at the athletics track, the height and weight of the students were measured with a portable Seca stadiometer 208 (Vogel & Halke, Hamburg, Germany) and with a portable Seca 762 scale (Vogel & Halke, Hamburg, Germany), respectively. Body mass index (BMI) was calculated using the classic formula: weight(kg)/height(m)^2^. Both measurements were taken 3 h after the last meal, with the participants dressed in light clothing without shoes. After that, brachial blood pressure was measured twice after at least 5 min of rest in a seated position, with an appropriately sized cuff placed on the right arm, using a validated digital electronic tensiometer (Omron M4, Omron Corp., Kyoto, Japan). To analyse physical activity profiles, the International Physical Activity Questionnaire (IPAQ) was employed [38]. In addition, sleep patterns were analysed using Pittsburg sleep quality questionnaire [36]. Both physical activity and sleep questionnaires were answered by self-reporting. Once participants had filled out the questionaries, they performed a Cooper’s 12 min run test [37]. The Cooper test was performed on a 400 m synthetic athletic track with the supervision of the research team. Before the test began, participants performed a 15 min warm-up, including running at low intensity, joint mobility, dynamic stretching, and progressive running sets. Subsequently, the participants carried out the classic test protocol, which consisted of covering the maximum possible distance for 12 min. Then, the total distance covered after 12 min by the experimental subjects was measured in metres. VO_2_ max was predicted using the following formula [39]: VO_2_ max (mL/kg/min) = (22.351 × distance covered in kilometres) − 11.288.

### 2.4. Statistical Analysis

A statistical analysis was carried out using the Statistical Package for the Social Sciences (SPSS) version 25.0 (SPSS Inc., Chicago, IL, USA). Descriptive statistics (mean and standard deviation) were calculated. Before using para-metric tests, the assumptions of normality and homoscedasticity were verified using the Kolmogorov–Smirnov test. 

An independent *t*-test was conducted to explore differences between groups in the analysed variables. For all procedures, a level of *p* ≤ 0.05 was selected to indicate statistical significance. 

## 3. Results

Table 1 presents the summary statistics for the results of anthropometric, physiological variables and sleep patterns in the LAPG and HAPG. No significant differences were found in body composition variables and in physical activity habits between groups. However, the HAPG group had significantly higher VO_2_ max values (LAPG = 40.32 ± 6.07; HAPG = 47.91 ± 6.89 mL/kg/min; *p* < 0.001) and lower diastolic blood pressure than the LAPG (LAPG = 72.44 ± 14.27; HAPG = 67.48 ± 13.50 mmHg; *p* < 0.01). Finally, a significantly lower value of insomnia caused by breathing problems was found in HAPG in comparison with LAPG (LAPG = 0.37 ± 0.8; HAPG = 0.13 ± 0.42 a.u.; *p* = 0.046). No significant differences were found in other sleep quality variables.

## 4. Discussion

The aim of the present study was to analyse body composition, cardiovascular function, sleep habits and physical activity factors related to the academic performance of university students. The initial hypothesis was partially confirmed since students with a higher academic performance presented a higher VO_2_ max than students with a lower academic performance, as well as lower diastolic blood pressure and insomnia levels caused by breathing problems.

VO_2_ max is the maximal rate of pulmonary oxygen uptake during the practice of a physical activity that requires sufficient muscle mass [40]. Furthermore, VO_2_ max has been highlighted as an inverse parameter related to cardiovascular disease, with lower rates of VO_2_ max being linked to a greater cardiovascular risk [41,42,43]. Lower VO_2_ max levels are also associated with a worse physical activity condition, as indicated by previous authors and seen in the lower VO_2_ max (maximum oxygen consumption capacity) and aerobic capacity demonstrated by subjects [44]. Regarding the influence of VO_2_ max and academic achievement, higher VO_2_ max rates were also found to be positively correlated with academic performance in university students [45], and our findings agree with these results since the high academic performance group showed greater VO_2_ max rates. This is explained by the fact that maximum oxygen consumption capacity correlates with better physical activity conditions and, consequently, with better academic achievement, likely due to the benefits of physical activity. These benefits may be explained by several factors. Firstly, these benefits could be justified by the presence of the brain-derived neurotrophic factor (BDNF), whose synthesis is enhanced by cardiorespiratory fitness and whose activity could improve academic performance [23,24]. Secondly, this difference in physical activity benefits may also be supported by the higher levels of exercise in the high academic performance group. Finally, this is also explained by the fact that, if there were higher rates of VO_2_ max, there would be greater O_2_ concentrations available to different tissues, including CNS, and this would improve prefrontal cortex functions due to increased oxygen availability, as previous authors have suggested [46]. 

Regarding blood pressure and academic performance, the low academic performance group showed significantly higher values of diastolic blood pressure. Nevertheless, we found several discrepancies around this issue, since no significant differences were found between academic performance and diastolic blood pressure in previous research [47].

Furthermore, previous research described how high values of blood pressure, without specifying if it was systolic or diastolic, were significantly associated with lower mean academic performance scores in school students studying some subjects [48]. The results of the present study were consistent with this study, and the difference between students’ ages—which may constitute a confounding factor, as well as the fact that our results for diastolic blood pressure could not been defined as clinically greater—was only significantly higher between both performance groups. Regarding systolic blood pressure, i previous researchers described how systolic blood pressure was significantly negatively associated with academic achievement [49], but no significant differences were found in the present study related to systolic blood pressure and academic performance. When contemplating all of these discrepancies, we suggest that more research is conducted on the relationship between blood pressure and academic success.

Regarding insomnia and its impact on academic performance, it has been widely reported that insomnia may significantly reduce academic achievement [50,51,52,53]. The results of the present study are consistent with these studies since those in the low academic performance group experienced more episodes of insomnia. In the present study, a positive association was found between lower academic performance and insomnia caused by breathing problems. This relationship was consistent with previous studies such as [54,55], where snoring or obstructive sleep apnoea were related to poorer academic performance. However, this finding simply reveals a relationship between the variables and does not establish the direction of causation or eliminate the influence of an intervening variable, some causal explanations are more likely than others. A likely explanation of the relationship between insomnia and academic achievement could be that poor sleep is associated with a lack of concentration and incapability to effectively operate during the day [56,57], which may have a negative impact on academic performance. Additionally, previous researchers also found that lower VO_2_ max rates were associated with worse sleep quality [58]. The results of the present study agree with this study since a positive relation was found between students in the low academic performance, who showed fewer VO_2_ max rates and insomnia problems. Thus, the fact that the low academic performance group showed poorer academic achievement may suggest a relationship between low rates in VO_2_ max, insomnia and academic performance. Nevertheless, previous researchers also pointed out that there is no association between academic achievement and insomnia problems [59]. This controversy may indicate that more studies are needed to establish this relationship.

### 4.1. Practical Applications

Physical activity is indicated as an important key factor of academic performance since it improves brain neurotrophic factors, brain development, and overall health status. 

Additionally, sleep habits have also been highlighted as important elements of academic achievements since successful sleep quality and time is related to better academic achievement.

Therefore, it is possible to consider the implementation of student programmes, through the utilization of seminars, talks and different communication routes, with the aim of providing advice and recommendations to help improve their exercise and sleep habits; consequently, they would be able to enhance their academic performance.

### 4.2. Limitation of the Study and Future Research Lines

The main limitation of the present research was the lack of biological measures of neurotrophin family proteins (BDNF) due to resource availability. Future research may address these issues, as it would improve the total knowledge of physical activity effect on academic performance. 

Additionally, another limitation of the study was that we did not consider the effect that stress may have on sleep disorders, considering it in a SUDS (Subjective Units of Distress) scale. Regarding academic performance parameters, only grades were considered as indicators of academic achievement, but it would be interesting to consider the use of different skills, such as communication, teamwork, creativity or critical thinking, in order to contemplate different aspects of the university experience. Future studies may contemplate these issues.

Moreover, another limitation of this study was its low number of participants, since increasing the study population may offer a better extrapolation of results to the general student population. Future research may contemplate increasing the number of participants.

The final limitation of the present study was that we did not consider gender in the study. Considering that the sample was so symmetrically distributed (49.8% males and 50.2% females), it would have been really interesting to see if the discovered differences and correlations are maintained by gender.

Future lines of research should consider the use of effective sleep habits and physical activity interventions that may enhance overall health status, as well as academic performance in students. 

## 5. Conclusions

We found a relevant correlation between academic performance and VO_2_ max, diastolic blood pressure and insomnia caused by breathing problems, showing that the low academic performance group had significantly higher diastolic blood pressure and insomnia levels, as well as significantly lower VO_2_ max scores. These results highlight the importance of applying different programmes that may improve these factors, especially those related to physical activity and sleep habits, with the aim of enhancing academic achievement.

## Figures and Tables

**Table 1:** Anthropometric and physiological variables and physical activity and sleep habits differences between low academic performance group (LAPG) and high academic performance group (HAPG).

					95% Confidence	
Variables	LAPG	HAPG	T	*p*-Value	Lower	Upper
Grade	3.72 ± 0.91	6.57 ± 1.03	−23.52	<0.01	−3.08	−2.60
Height (cm)	176.37 ± 8.05	176.2 ± 7.31	0.177	0.860	−1.71	2.04
Weight (kg)	73.95 ± 11.17	73.92 ± 11.67	0.018	0.986	−2.76	2.81
Body mass index (kg/m^2^)	23.70 ± 2.81	23.72 ± 3.03	−0.036	0.971	−0.73	0.70
Physical activity (hours per week)	7.71 ± 4.58	7.74 ± 4.27	−0.06	0.953	−1.11	1.04
Systolic blood pressure (mmHg)	116.20 ± 19.23	114.67 ± 19.88	0.625	0.533	−3.30	6.38
Diastolic blood pressure (mmHg)	72.44 ± 14.27	67.48 ± 13.50	2.84	<0.01	1.52	8.40
VO_2_ max (mL/kg/min)	40.32 ± 6.07	47.91 ± 6.89	−9.313	<0.01	−9.19	−5.98
Time to fall asleep (min)	28.24 ± 26.16	24.22 ± 23.14	0.826	0.411	−5.64	13.69
Estimated hours of sleep	7.08 ± 1.03	7.42 ± 1.10	−1.567	0.120	−0.76	0.09
Insomnia caused by not falling asleep	1.17 ± 0.89	1.32 ± 0.93	−0.83	0.408	−0.516	0.211
Insomnia caused by night waking	1.05 ± 0.97	1.02 ± 0.97	0.17	0.864	−0.352	0.42
Insomnia caused by incontinence	0.93 ± 1.03	0.83 ± 0.97	0.482	0.631	−0.3	0.490
Insomnia caused by breathing problems	0.37 ± 0.8	0.13 ± 0.42	2.02	0.046	0.005	0.477
Insomnia caused by coughing and snoring	0.33 ± 0.73	0.34 ± 0.81	−0.85	0.930	−0.326	0.299
Insomnia caused by cold feeling	0.63 ± 0.83	0.63 ± 0.76	0.021	0.983	−0.308	0.315
Insomnia caused by hot feeling	0.85 ± 0.93	0.66 ± 0.80	1.15	0.252	−0.142	0.537
Insomnia caused by nightmares	0.66 ± 0.76	0.69 ± 0.79	−0.185	0.853	−0.339	0.281
Insomnia caused by pain feeling	0.34 ± 0.61	0.340.60	0.025	0.98	−0.235	0.241

## Data Availability

Not applicable.
